# Close of Volume, Etc.

**Published:** 1879-04

**Authors:** 


### EDITORIAL, ETC.

With this number closes the twelfth volume of the third series of this Journal. It is hoped and expected that its future will be encouraging, and that its friends will see to it that more original matter of a truly original character comes to its pages than heretofore. The premium offered by the Publishers makes it lower in price than the lowest, and the Editors hope to give it in the future more of diligent and discriminating attention than heretofore. The common phase that a journal is published to "supply a *felt want*," is often quoted in defence of the origination of a new periodical; it should apply with force to this, the only one published south of Philadelphia, and indeed the only one in the South, where reside many of the foremost men of the profession, and from which for many years came the chief impulses which forwarded the state of things that made dentistry a liberal profession.

Those managing the AMERICAN JOURNAL OF DENTAL SCIENCE again appeal to the profession throughout the country to put the result of their observations on paper, and let the world see them. Notoriously, some of the best men never write. This is all wrong; charity for their fellows, and interest in a profession to which the best energies of life are given should prompt the recording of facts and, if you please, the generalization therefrom of theories.

Some notable things have transpired during the past year. The "New Departure" theories, concerning plastic, and especially concerning the use of amalgam fillings, are evidently moulding professional opinions and the opinions of our patients also. That more amalgam is used, is part due to the knowledge of its value on the part of the unprofessional public. Perhaps this is well. Certainly in these times when financial difficulties make the insertion of the most costly gold fillings an impossibility for the mass of the patients who call for treatment, and when a large fee means the loss of a valuable organ, it is well that the more scientific side of the subject is being examined into, and the question at least lifted out of the domain of empiricism.

The consolidation of the two Dental Colleges, the Maryland Dental College with the old Baltimore College of Dental Surgery, is an event which seems to have been demanded by the times, and is heralded as a sign of greater unity, and, as a consequence, greater premised good. Whether the colleges are "making too many dentists," is a question for debate in associations, but the most casual observer must see that the wholesale multiplication of these institutions can only result in harm; weakening the influence of all and making ungenerous and unfriendly competition a necessary result. It was earnestly felt by the friends of both institutions that some move should be made which would condense into one focus the influence divided in the South between two colleges. The friends of both seem pleased, and the promise is made that the future shall show that those who desire a standard of efficiency on the part of graduates equal to the demands of the times shall not be disappointed.

Let all then, only looking back on the past and on the jarrings which were painful, gather useful lessons, and with determinations for the most zealous efforts for the future, try to help advance the cause of dental education, knowing that ignorance is the great obstacle in the way of the progress of any profession. H.

---

